# Predictive factors for unfavorable outcomes of tuberculous pericarditis in human immunodeficiency virus–uninfected patients in an intermediate tuberculosis burden country

**DOI:** 10.1186/s12879-016-2062-5

**Published:** 2016-11-29

**Authors:** In Young Jung, Young Goo Song, Jun Yong Choi, Moo Hyun Kim, Woo Yong Jeong, Dong Hyun Oh, Yong Chan Kim, Je Eun Song, Eun Jin Kim, Ji Un Lee, Su Jin Jeong, Nam Su Ku, June Myung Kim

**Affiliations:** Department of Internal Medicine and AIDS Research Institute, Yonsei University College of Medicine, 50-1 Yonsei-ro, Seodaemun-gu, 120-752 Seoul Republic of Korea

**Keywords:** Tuberculous pericarditis, Non-HIV, Unfavorable outcome

## Abstract

**Background:**

In areas where *Mycobacterium tuberculosis* is endemic, tuberculosis is known to be the most common cause of pericarditis. However, the difficulty in diagnosis may lead to late complications such as constrictive pericarditis and increased mortality. Therefore, identification of patients at a high risk for poor prognosis, and prompt initiation of treatment are important in the outcome of TB pericarditis. The aim of this study is to identify the predictive factors for unfavorable outcomes of TB pericarditis in HIV-uninfected persons in an intermediate tuberculosis burden country.

**Methods:**

A retrospective review of 87 cases of TB pericarditis diagnosed at a tertiary referral hospital in South Korea was performed. Clinical characteristics, treatment outcomes, complications during treatment, duration of treatment, and medication history were reviewed. Unfavorable outcome was defined as constrictive pericarditis identified on echocardiography performed 3 to 6 months after initial diagnosis of TB pericarditis, cardiac tamponade requiring emergency pericardiocentesis, or death. Predictive factors for unfavorable outcomes were identified.

**Results:**

Of the 87 patients, 44 (50.6%) had unfavorable outcomes; cardiac tamponade (*n* = 36), constrictive pericarditis (*n* = 18), and mortality (*n* = 4). 14 patients experienced both cardiac tamponade and constrictive pericarditis. During a 1 year out-patient clinic follow up, 4 patients required repeat pericardiocentesis and pericardiectomy was performed in 0 patients. In the multivariate analysis, patients with large amounts of pericardial effusion (*P* = .003), those with hypoalbuminemia (*P* = .011), and those without cardiovascular disease (*P* = .011) were found to have a higher risk of unfavorable outcomes.

**Conclusion:**

HIV-uninfected patients with TB pericarditis are at a higher risk for unfavorable outcomes when presenting with low serum albumin, with large pericardial effusions, and without cardiovascular disease.

**Electronic supplementary material:**

The online version of this article (doi:10.1186/s12879-016-2062-5) contains supplementary material, which is available to authorized users.

## Background

Tuberculosis is a globally important infectious disease with a high mortality and morbidity, with 1.5 million deaths among the human immunodeficiency virus (HIV)–negative and 0.52 million deaths among the HIV–positive population, and with 9.4 million new cases reported each year worldwide in 2014 [1]. In areas where *Mycobacterium tuberculosis* is endemic, tuberculosis is known to be the most common cause of pericarditis [2]. Although the incidence of tuberculosis has decreased in many Western countries, tuberculous (TB) pericarditis is still seen in many patients in countries where tuberculosis is still an endemic disease [3]. According to a study conducted in Tanzania, TB pericarditis is also more likely to occur in HIV-infected patients, whereas HIV-uninfected patients are likely to have other etiologies of pericardial effusions [4].

Symptoms of TB pericarditis are generally nonspecific, with an insidious onset [5]. The frequency of common symptoms varies; however, cough, dyspnea, chest pain, night sweat, orthopnea, and weight loss are generally the most frequent symptoms [6, 7]. The diagnosis of TB pericarditis can be made through the identification of the tubercle bacillus from pericardial fluid; however, invasive diagnostic pericardial biopsy is not an easy process to perform [8]. Anti-TB therapy is effective in reducing mortality among HIV-uninfected persons to 8–17% [9]. A retrospective study conducted in Birmingham showed that antituberculosis medication reduced the likelihood of developing constrictive pericarditis to 10–20% [7]. However, the difficulty in diagnosis may lead to late complications such as constrictive pericarditis and increased mortality [5]. Therefore, identification of patients at a high risk for poor prognosis, and prompt initiation of treatment are important in the outcome of TB pericarditis.

We conducted this study to identify the predictive factors for the unfavorable outcomes of TB pericarditis in HIV-uninfected patients in an intermediate tuberculosis burden country.

## Methods

### Study population

We studied 166 patients with TB pericarditis diagnosed at a tertiary referral hospital in South Korea during an 11-year period (January 2005 through December 2015). Seventy-nine patients with diagnostic error (*n* = 11), missing laboratory data (*n* = 60), loss to treatment follow-up (*n* = 7), or concomitant HIV infection (*n* = 1) were excluded. A total of 87 patients were reviewed. Patients who were 18 years old or older who had been confirmed to have probable or definite TB pericarditis were included.

### Collected data

The following information were collected: demographic data, comorbidities, initial presenting symptoms, laboratory results, echocardiographic findings, pericardial biopsy results, pericardial fluid smear microscopy for acid fast bacilli, pericardial culture for *M. tuberculosis*, antimicrobial susceptibility of *M. tuberculosis* strains isolated from the patients, pharmaceutical formulation use, steroid use, and duration of treatment. Patient follow-up was based on out-patient clinical visits.

### Definitions

Definite TB pericarditis was defined as a diagnosis confirmed on a pericardial sample based on finding acid and alcohol fast bacilli on microscopy, positive microbiological culture for *M. tuberculosis*, presence of caseating granulomata on histology, or positive nucleic acid test (fluid or tissue) [10].

Probable TB pericarditis was defined as the presence of a lymphocytic pericardial exudate with elevated adenosine deaminase (ADA) activity ≥40 IU/L [10]. Detailed information on definitions of definite and probable TB pericarditis is provided in Additional file 1: Table S1.

Unfavorable outcome was defined as constrictive pericarditis identified on echocardiography performed 3 to 6 months after initial diagnosis of TB pericarditis, cardiac tamponade requiring emergency pericardiocentesis, or death [10].

Cardiovascular disease was defined in patients with a history of hypertension, stable angina, and congestive heart failure. The amount of pericardial effusion was categorized into small (50–100 mL), moderate (100–500 mL), or large (>500 mL) [11]. QuantiFERON-TB Gold In-Tube assay was used for interferon-gamma (IFN-γ) release assay (IGRA) diagnosis. The IFN-γ levels (IU/mL) were measured, and the results were reported as positive, negative, or indeterminate. Hypoalbuminemia was defined as serum albumin <3.0 g/dL. Body mass index was calculated as the weight in kilograms divided by the square of height in meters [12]. Red blood cell (RBC) distribution width was defined as the index of RBC size heterogeneity and RBC distribution width [13].

### Statistical analysis

All factors were compared between the unfavorable and favorable outcome groups. Categorical variables are presented as numbers and percentages. Continuous variables are expressed as mean ± standard deviation, unless otherwise indicated. Categorical variables were compared by using χ^2^ analysis, and continuous variables with normal distributions were compared by using Student’s *t*-test. We used the Mann–Whitney *U*-test to compare the continuous variables with a skewed distribution. Multivariate analysis was performed through logistic regression to identify factors that independently and significantly affected the outcome. All categories (large pericardial effusions, dyspnea, male sex, albumin, and cardiovascular disease) were calculated as a percentage with the 95% confidence interval (CI). All statistical tests were performed by using IBM SPSS software for Windows, version 20. *P*-values < 0.05 were considered statistically significant.

## Results

As shown in Table 1, of the 87 cases reviewed, 44 (50.6%) patients had unfavorable outcome. Of these patients, 36 patients received pericardial effusion drainage due to cardiac tamponade, 18 patients had constrictive pericarditis, and mortality in 4 patients. 14 patients experienced both cardiac tamponade and constrictive pericarditis. During a 1 year out-patient clinic follow-up, 4 patients required repeat pericardiocentesis, and pericardiectomy was performed in 0 patients. The causes of mortality were acute heart failure due to stress-induced cardiomyopathy, and cardiac tamponade.

**Table 1:** The baseline characteristics of the patients

Characteristic	Favorable (*n* = 43)	Unfavorable (*n* = 44)	*P*-value
Age (years)	66.5 ± 18.2	67.1 ± 15.5	.873^a^
Male, n (%)	15 (34.9)	25 (56.8)	.040^b^
BMI	22.8 ± 4.9	22.9 ± 3.2	.871^a^
Underlying diseases, yes (%)			
Solid cancer	2 (4.7)	4 (9.1)	.676^b^
Hematologic malignancy	0 (0.0)	3 (6.8)	.241^b^
Hypothyroidism	0 (0.0)	2 (4.5)	.504^b^
Cardiovascular disease	24 (55.8)	15 (34.1)	.042^b^
Old tuberculosis	6 (14.0)	8 (18.2)	.592^b^
Chronic renal disease	4 (9.3)	1 (2.3)	.202^b^
Chronic liver disease	0 (0.0)	3 (6.8)	.241^b^
Chronic lung disease	3 (7.0)	5 (11.4)	.713^b^
Autoimmune disease	1 (2.3)	4 (9.1)	.360^b^
DM	7 (16.3)	12 (27.3)	.300^b^
Laboratory findings			
Leukocyte count (PE)	2456 (1020–3500)	1110 (225–3420)	.205^c^
ADA	63 (48–103)	85 (46–125)	.228^c^
Albumin, g/dL	3.6 ± 0.6	3.3 ± 0.7	.047^a^
Hypoalbuminemia, n (%)^e^	7 (16.3)	17 (38.6)	.020^b^
Symptoms			
Dyspnea	27 (62.8)	37 (84.1)	.024^b^
Chest pain	17 (39.5)	16 (36.4)	.761^b^
Fever	14 (32.6)	8 (18.2)	.123^b^
Palpitation	2 (4.7)	7 (15.9)	.157^b^
Duration, days	7 (5–21)	14 (7–28)	.433^c^
PE amount			.023^d^
Small	8 (18.6)	4 (9.1)	.198^b^
Moderate	12 (27.9)	5 (11.4)	.062^b^
Large	23 (53.5)	35 (79.5)	.010^b^
Concomitant pulmonary tuberculosis	6 (14.0)	4 (9.1)	.521^b^
Origin			
Lung	8 (18.6)	6 (13.6)	.528^b^
Reactivation and inapparent	35 (81.4)	38 (86.4)	
Hospital stay, days	12.2 ± 11.0	14.3 ± 10.5	.348^a^
Antituberculosis medication			
Duration, months	6.4 ± 2.3	6.6 ± 3.3	.776^a^
Steroid			
Users, yes (%)	19 (44.2)	22 (50.0)	.587^b^
Duration, weeks	12.1 ± 7.4	11.6 ± 7.3	.808^a^

Data are expressed as the mean ± standard deviation or number (percentage)

*BMI* body mass index, *DM* diabetes mellitus, *PE* pericardial effusion, *ADA* adenosine deaminase

^a^Student’s *t*-test

^b^Pearson’s χ-test

^c^Mann–Whitney *U*-test, median (interquartile range)

^d^Linear-by-linear association

^e^defined as serum albumin concentration below 3.0 g/dL

There was a male predominance in patients with unfavorable outcome compared with those with favorable outcome (56.8% vs. 34.9%, *P* = .040). Other baseline characteristics of underlying diseases such as solid organ cancer, hematologic malignancies, hypothyroidism, old pulmonary tuberculosis, chronic renal disease, chronic liver disease, chronic lung disease, autoimmune disease, and diabetes mellitus did not show any difference between outcomes. On the other hand, patients without a history of cardiovascular disease were found to have a higher risk of unfavorable outcomes (34.1% vs. 55.8%, *P* = .042).

Table 1 shows the laboratory results of the study participants at the time of admission. Patients presenting with a low albumin level at the time of admission tended to have unfavorable outcomes (3.3 ± 0.7 vs. 3.6 ± 0.6, *P* = .047). Hypoalbuminemia defined as serum albumin lower than 3.0 g/dL had higher risk of unfavourable outcome (38.6% vs. 16.3%, *P* = .020). Pericardial ADA levels did not show a statistical significance between the two groups (85 vs. 63, *P* = .986).

Table 1 describes the clinical symptoms and findings of the study participants. Dyspnea was the most common presenting symptom, and the presence of dyspnea showed a correlation to a higher risk of unfavorable outcomes (84.1% vs. 62.8%, *P* = .024). Patients who presented with large amounts of pericardial effusion, exceeding 500 mL on the echocardiogram, showed a higher risk for unfavorable outcomes (79.5% vs. 53.5%, *P* = .010). Concomitant pulmonary tuberculosis was seen in 6 (14%) patients with favorable outcomes and 4 (9.1%) patients with unfavorable outcomes, with no significant difference (*P* = .521). The mean duration of symptom onset was 7 days in the favorable group and 14 days in the unfavorable group, showing that patients with unfavorable outcomes were admitted for hospital care earlier; however, the difference was not statistically significant.

All strains of *M. tuberculosis* isolated from the patients were antimicrobial susceptible strains in this study. Anti-TB medication (standard isoniazid, rifampin, ethambutol, and pyrazinamide for 2 months followed by maintenance 4 months of isoniazid, rifampin, and ethambutol) was taken for a duration of 6.4 ± 2.3 months in the favorable outcome group and 6.6 ± 3.3 months in the unfavorable outcome group, with no significant difference (*P* = .776). Steroids were administered in 19 (44.2%) patients with favorable outcomes and in 22 (50%) patients with unfavorable outcomes (*P* = .587). The mean duration of steroid use (prednisolone 60 mg/day for four weeks, followed by 30 mg/day for four weeks, 15 mg/day for two weeks, and 5 mg/day for 1 week [14]) was 12.1 ± 7.4 weeks in the favorable outcome group and 11.6 ± 7.3 weeks in the unfavorable outcome group (*P* = .808). In the Kaplan Meier analysis, patients with cardiac tamponade had a lower rate of survival compared to patients with favorable outcomes (mean survival days: 1 day vs. 28 days, *P* < .001).

In the multivariate analysis, a large amount of pericardial effusion on echocardiogram (odds ratio [OR] 5.974, 95% CI 1.811–19.703, *P* = .003), hypoalbuminemia (OR 4.905, 95% CI 1.443–16.667, *P* = .011), and absence of underlying cardiovascular disease (OR 0.255, 95% CI 0.089–0.733, *P* = .011) were shown to be independent predictive factors for unfavorable outcomes (Table 2).

**Table 2:** Multivariate analysis of predictive factors for unfavorable outcomes in patients with tuberculous pericarditis

Variables	OR (95% CI)	*P*-value
Large PE	5.974 (1.811–19.703)	.003
Cardiovascular disease	0.255 (0.089–0.733)	.011
Hypoalbuminemia^a^	4.905 (1.443–16.667)	.011
Dyspnea	2.792 (0.852–9.145)	.090
Male	2.063 (0.745–5.713)	.163

*OR* odds ratio, *CI* confidence interval, *PE* pericardial effusion

^a^defined as serum albumin concentration below 3.0 g/dL

## Discussion

The infection of *M. tuberculosis* in the pericardium usually occurs through the retrograde lymphatic spread of the infection from the lungs, mediastinum structures, and adjacent lymph nodes, or through the hematogenous spread from the spine or from genitourinary infection [15]. The pathogenesis of TB pericarditis is a delayed hypersensitivity response induced by the activation of lymphocytes releasing lymphocytokines that activate macrophages, leading to granuloma formation and pericardial effusion [16]. The histopathological pattern is mostly affected by the immune state, and HIV-infected persons with severely depleted CD4 lymphocytes present with less granuloma on biopsy [17].

While there is no difference in the sex–age pattern of the disease in industrialized countries, the disease rate in adult men exceeds those in women in developing countries [18]. The prevalence of *M. tuberculosis* infection in developing countries is similar to these findings, with a male predominance in patients older than 15 years [18]. The difference in the tuberculosis prevalence and sex is explained by the fact that women in developing countries have a passive attitude about visiting health-care facilities owing to cultural reasons [19]. The result of our study shows that male sex confers a higher risk for unfavorable outcomes.

The normal amount of pericardial fluid is 10–15 mL between the pericardial layers, and acts as a lubricant [20]. An inflammatory process of any cause results in an increased production of pericardial fluid [20, 21]. In previous studies, patients with hemodynamic compromise have large amounts of pericardial effusion on echocardiogram and are often associated with a higher mortality rate [2, 22]. Hemodynamically significant pericardial effusions leading to cardiac tamponade are commonly apparent with dyspnea [6, 23]. Dyspnea on exertion is a key symptom of acute pericardial tamponade that requires emergency pericardiocentesis and has a high risk for mortality [24]. Choi et al. reported that patients who were initially more symptomatic, and had echogenic pericardial effusions tended to require pericardiectomy more often, and had more advanced disease [25]. From these results, it can be presumed that patients with large amounts of pericardial effusion are likely to experience dyspnea leading to hemodynamic compromise, which is correlated with unfavorable outcomes.

In this study, the presence of cardiovascular disease was a predictive factor for favorable outcomes. In this study, patients with an underlying cardiovascular disease were regularly being followed by the hospital, which may have resulted in the earlier detection of symptoms or provided the availability of earlier medical care, compared with patients who did not conduct regular hospital visits. For these reasons, patients with an underlying cardiovascular disease have a low risk for unfavorable outcomes. However, further studies for general applicability, and to confirm the validity of this assertion is needed.

In a study conducted by Reuter et al., patients uninfected with HIV had significantly higher serum protein and globulin levels than HIV-infected tuberculosis patients [8]. Although there was no statistical significance in serum albumin levels, the serum albumin/globulin ratios showed significantly lower results in the HIV-uninfected TB pericarditis group [8]. One study in India identified serum albumin concentrations <3.2 g/dL as a risk factor for increased mortality in HIV-infected patients with tuberculosis [26]. However, there have been no studies on serum albumin level and its prognostic value in HIV-uninfected patients with TB pericarditis. The significance of this study is that it shows serum albumin as an independent variable predictive of unfavorable outcome in HIV-uninfected patients.

ADA is considered a marker for cellular immunity and is associated with the differentiation of lymphocytes [27]. A cutoff value of 40 U/L in the pericardial fluid is considered to have a specificity and sensitivity of 72% and 89% in diagnosis of TB pericarditis, respectively [28]. A median ADA of 97 U/L in the pericardial fluid was seen in the patients of this study.

The South Korean population receives routine Bacille Calmette-Guérin (BCG) vaccination after birth, and again at age 12 or 13 years if the child proves to be a tuberculin skin test (TST) nonresponder. Because BCG vaccination shows cross-reactivity with the result of the TST, we considered serum IGRA to be more appropriate than the TST for the diagnosis of latent tuberculosis [29]. In a study by Burgess et al., the measurement of pericardial fluid IGRA with a cutoff level of >200 pg/L was highly specific (100%) [30]. Future studies in larger sample numbers may confirm the use of pericardial effusion IGRA measurement as a promising method for the rapid diagnosis of TB pericarditis.

Anti-TB therapy reduces the incidence of mortality and constrictive pericarditis [7, 31]. The treatment duration and regimen approach in HIV-uninfected patients is the same as those for pulmonary tuberculosis [32, 33]. The 6-month treatment regimen has similar effectiveness to that of longer treatment duration, and is generally recommended in susceptible tuberculosis cases [33]. Although the antimycobacterial treatment duration was slightly longer in the unfavorable group, it did not have any statistical difference with a median treatment duration of 6 months (6.6 ± 3.3 vs. 6.4 ± 2.3, *P* = .776).

In several prior studies, the use of corticosteroids significantly reduced the incidence of constrictive pericarditis, and thereby patients at high risk of progression to constrictive pericarditis are suggested to receive an adjunctive use of corticosteroids with a recommended starting dose of 60 mg/day tapered during 11 weeks [10, 14, 34]. Another 10 years follow-up study by Strang et al. described that prednisolone reduced the risk of death due to pericarditis, and the need for repeat pericardiocentesis in patients with constrictive pericarditis or pericardial effusion [35, 36]. In our study, among the 40 patients with effusion or constrictive pericarditis, 21 were treated with steroids during a mean duration of 11 weeks but did not show any association to unfavorable outcomes. Hence, there was a difference in outcomes compared to previous clinical trials. However, these previous trials had relatively small samples, and a recent randomized trial of 1400 patients (431 whom were HIV-negative) by Mayosi et al. demonstrated similar results to our study, that routine use of adjuvant corticosteroids did not have any significant effect on the primary outcomes of death and cardiac tamponade [10].

This study has several limitations. First, this study was conducted retrospectively. Therefore, information on nutritional status, which could influence the treatment outcome, was not available. Second, only a small number of patients were included. Third, while it is plausible that routine follow up for cardiovascular disease may have enhanced earlier detection of symptoms, the explanation for this association may have limitations for general applicability. Finally, this study was performed in a country with an intermediate tuberculosis prevalence. Future multicenter prospective studies in countries with a diverse tuberculosis epidemiology are needed to confirm the predictive factors for unfavorable outcomes in HIV-uninfected persons.

## Conclusion

HIV-uninfected patients with TB pericarditis are at a higher risk for unfavorable outcomes when presenting with hypoalbuminemia, with large pericardial effusion, and without cardiovascular disease. This study may prompt clinicians to perform early diagnosis and treatment in patients with these risk factors, which may lead to a decrease in mortality and late complications such as constrictive pericarditis.

## Additional file

Additional file 1: Table S1. Definition of diagnostic categories. (DOCX 21 kb)

## Abbreviations

ADA: Adenosine deaminase
BCG: Bacille Calmette-Guérin
CI: Confidence interval
HIV: Human immunodeficiency virus
IFN-γ: Interferon-gamma
IGRA: Interferon gamma release assay
OR: Odds ratio
RBC: Red blood cell
TB: Tuberculous
TST: Tuberculin skin test
