# Supplementary material for: Predictive factors for unfavorable outcomes of tuberculous pericarditis in human immunodeficiency virus–uninfected patients in an intermediate tuberculosis burden country
Source: BMC Infect Dis. 2016 Nov 29;16:719. doi: 10.1186/s12879-016-2062-5 (PMC5129391; doi:10.1186/s12879-016-2062-5)
Supplement: Additional file 1: Table S1. — Definition of diagnostic categories. (DOCX 21 kb) [file 12879_2016_2062_MOESM1_ESM.docx]

| **Table S1: Definition of diagnostic categories [**[**1**](#_ENREF_1)**]** | | |
| --- | --- | --- |
| Category | Definite TB pericarditis | Probable TB pericarditis |
| Definitions | Acid and alcohol fast bacilli on microscopy  or  Positive microbiological culture for *M.* *tuberculosis*  or  Caseating granulomata on histology  or  Positive nucleic acid test (fluid or tissue) | Lymphocytic pericardial exudate with elevated adenosine deaminase (ADA) activity ≥40 IU/L |

TB, tuberculosis; M. tuberculosis, *Mycobacterium tuberculosis*; PCR, polymerase chain reaction

**SUPPLEMENTARY REFERENCES**

1. Mayosi BM, Ntsekhe M, Bosch J, Pandie S, Jung H, Gumedze F, Pogue J, Thabane L, Smieja M, Francis V *et al*: **Prednisolone and Mycobacterium indicus pranii in tuberculous pericarditis**. *The New England journal of medicine* 2014, **371**(12):1121-1130.
